# Supplementary material for: Comparative genomics provides new insights into the diversity, physiology, and sexuality of the only industrially exploited tremellomycete: Phaffia rhodozyma
Source: BMC Genomics. 2016 Nov 9;17:901. doi: 10.1186/s12864-016-3244-7 (PMC5103461; doi:10.1186/s12864-016-3244-7)
Supplement: Additional file 6: — List of orphan genes with links to PFAM (related to Additional file 1: Table S1). (ZIP 1428 kb) [file 12864_2016_3244_MOESM6_ESM.zip › PFAM_UNIQUE/G03801_P.html]

#                                                                            --- full sequence --- -------------- this domain -------------   hmm coord   ali coord   env coord
  
# target name        accession   tlen query name           accession   qlen   E-value  score  bias   #  of  c-Evalue  i-Evalue  score  bias  from    to  from    to  from    to  acc description of target
  
#------------------- ---------- ----- -------------------- ---------- ----- --------- ------ ----- --- --- --------- --------- ------ ----- ----- ----- ----- ----- ----- ----- ---- ---------------------
  
G03801\_P             -            848 AAA\_13               PF13166.1    712    0.0027   14.9   0.5   1   1   2.7e-05    0.0043   14.2   0.5   333   467   346   483   322   516 0.70 - AAA domain  
G03801\_P             -            848 AAA\_27               PF13514.1   1111       1.1    5.5  12.4   1   1    0.0061       1.5    5.0  12.4   743   830   384   472   342   532 0.54 - AAA domain  
G03801\_P             -            848 APG6                 PF04111.7    315      0.27    8.9  15.7   1   2     0.013       2.3    5.8   1.4    42   132   341   431   329   434 0.85 - Autophagy protein Apg6  
G03801\_P             -            848 APG6                 PF04111.7    315      0.27    8.9  15.7   2   2    0.0077       1.4    6.6  14.0    17   129   385   512   376   531 0.60 - Autophagy protein Apg6  
G03801\_P             -            848 Ax\_dynein\_light      PF10211.4    189      0.12   10.8   5.7   1   1   0.00053      0.32    9.5   5.7   114   179   420   484   379   492 0.62 - Axonemal dynein light chain  
G03801\_P             -            848 Bap31                PF05529.7    192       7.5    4.6   7.9   1   3       1.3   1.3e+03   -2.7   0.0    25    78    12    66    10    83 0.61 - B-cell receptor-associated protein 31-like  
G03801\_P             -            848 Bap31                PF05529.7    192       7.5    4.6   7.9   2   3      0.16   1.6e+02    0.3   0.0    22    45   210   233   207   238 0.85 - B-cell receptor-associated protein 31-like  
G03801\_P             -            848 Bap31                PF05529.7    192       7.5    4.6   7.9   3   3    0.0041       4.1    5.5   2.8   120   178   365   450   357   483 0.69 - B-cell receptor-associated protein 31-like  
G03801\_P             -            848 BicD                 PF09730.4    717       5.6    3.8   9.9   1   1     0.064        15    2.5   9.9   287   420   350   486   341   529 0.56 - Microtubule-associated protein Bicaudal-D  
G03801\_P             -            848 CCDC144C             PF14915.1    305       0.3    8.6  12.3   1   3   0.00082      0.55    7.8   0.3    83   113   341   371   331   385 0.85 - CCDC144C protein coiled-coil region  
G03801\_P             -            848 CCDC144C             PF14915.1    305       0.3    8.6  12.3   2   3    0.0049       3.2    5.2   9.5   198   301   347   449   346   454 0.87 - CCDC144C protein coiled-coil region  
G03801\_P             -            848 CCDC144C             PF14915.1    305       0.3    8.6  12.3   3   3     0.019        12    3.3   6.6    49   109   391   451   382   483 0.78 - CCDC144C protein coiled-coil region  
G03801\_P             -            848 CENP-F\_leu\_zip       PF10473.4    140       6.5    5.3  18.1   1   2    0.0042       0.8    8.2   2.3    36   108   347   419   342   429 0.80 - Leucine-rich repeats of kinetochore protein Cenp-F/LEK1  
G03801\_P             -            848 CENP-F\_leu\_zip       PF10473.4    140       6.5    5.3  18.1   2   2       1.2   2.3e+02    0.3  10.7    17    81   419   483   414   501 0.57 - Leucine-rich repeats of kinetochore protein Cenp-F/LEK1  
G03801\_P             -            848 COG2                 PF06148.6    133      0.16   10.5   7.9   1   2   0.00064      0.25    9.9   0.4    66   118   346   398   345   421 0.82 - COG (conserved oligomeric Golgi) complex component, COG2  
G03801\_P             -            848 COG2                 PF06148.6    133      0.16   10.5   7.9   2   2     0.068        27    3.3   2.2    65   114   422   471   418   482 0.78 - COG (conserved oligomeric Golgi) complex component, COG2  
G03801\_P             -            848 DMPK\_coil            PF08826.5     61    0.0035   15.9   4.9   1   2       3.7   5.5e+03   -4.0   0.0    44    60   347   356   345   358 0.53 - DMPK coiled coil domain like  
G03801\_P             -            848 DMPK\_coil            PF08826.5     61    0.0035   15.9   4.9   2   2   2.5e-06    0.0038   15.8   3.0    17    59   404   446   396   448 0.89 - DMPK coiled coil domain like  
G03801\_P             -            848 DUF2353              PF09789.4    319       4.8    5.0   9.7   1   1     0.015       7.7    4.3   9.7    10   124   378   492   369   529 0.65 - Uncharacterized coiled-coil protein (DUF2353)  
G03801\_P             -            848 DUF3584              PF12128.3   1201     0.095    8.7  10.1   1   1     0.001      0.14    8.1  10.1   581   683   377   477   346   530 0.70 - Protein of unknown function (DUF3584)  
G03801\_P             -            848 DUF4407              PF14362.1    301     0.069   10.7   2.6   1   1   0.00046      0.17    9.5   2.6   147   232   385   475   258   485 0.77 - Domain of unknown function (DUF4407)  
G03801\_P             -            848 DUF724               PF05266.9    190       2.3    6.4   9.4   1   2   7.2e-05     0.072   11.4   1.7   101   177   372   451   349   455 0.78 - Protein of unknown function (DUF724)  
G03801\_P             -            848 DUF724               PF05266.9    190       2.3    6.4   9.4   2   2      0.55   5.5e+02   -1.3   1.1   104   182   458   533   456   537 0.70 - Protein of unknown function (DUF724)  
G03801\_P             -            848 DUF745               PF05335.8    188       1.6    6.9   6.1   1   2     0.044        33    2.6   0.4   107   158   347   398   339   409 0.74 - Protein of unknown function (DUF745)  
G03801\_P             -            848 DUF745               PF05335.8    188       1.6    6.9   6.1   2   2    0.0034       2.6    6.2   1.5    81   140   405   464   393   483 0.77 - Protein of unknown function (DUF745)  
G03801\_P             -            848 DUF869               PF05911.6    769   0.00014   18.9   8.4   1   1   7.9e-07   0.00021   18.4   8.4   529   726   263   479   118   492 0.66 - Plant protein of unknown function (DUF869)  
G03801\_P             -            848 DUF972               PF06156.8    107      0.13   11.3   8.5   1   2       5.4   1.2e+03   -1.5   0.0    40    58   346   364   341   389 0.55 - Protein of unknown function (DUF972)  
G03801\_P             -            848 DUF972               PF06156.8    107      0.13   11.3   8.5   2   2   5.5e-05     0.013   14.5   2.4     4    85   394   484   391   489 0.89 - Protein of unknown function (DUF972)  
G03801\_P             -            848 ERM                  PF00769.14   246         7    4.8  20.7   1   1     0.013       1.9    6.6  14.3     3   123   347   470   346   480 0.84 - Ezrin/radixin/moesin family  
G03801\_P             -            848 EzrA                 PF06160.7    560       1.1    6.1   4.7   1   1    0.0026       1.7    5.4   4.7   350   417   383   450   343   483 0.61 - Septation ring formation regulator, EzrA  
G03801\_P             -            848 FlgN                 PF05130.7    143         5    6.0   8.6   1   1     0.019        13    4.7   8.6     4   104   352   449   350   454 0.86 - FlgN protein  
G03801\_P             -            848 GAS                  PF13851.1    201       1.7    6.5  11.9   1   2     0.089        17    3.2   0.0    67   128   351   412   342   417 0.86 - Growth-arrest specific micro-tubule binding  
G03801\_P             -            848 GAS                  PF13851.1    201       1.7    6.5  11.9   2   2     0.034       6.5    4.5   9.0    34   122   412   500   408   521 0.88 - Growth-arrest specific micro-tubule binding  
G03801\_P             -            848 GOLGA2L5             PF15070.1    617       4.8    4.2   9.5   1   3      0.89   4.8e+02   -2.4   0.0    68    98   329   360   316   381 0.67 - Putative golgin subfamily A member 2-like protein 5  
G03801\_P             -            848 GOLGA2L5             PF15070.1    617       4.8    4.2   9.5   2   3     0.012       6.3    3.8   5.8    36   103   384   449   364   486 0.77 - Putative golgin subfamily A member 2-like protein 5  
G03801\_P             -            848 GOLGA2L5             PF15070.1    617       4.8    4.2   9.5   3   3     0.062        34    1.4   0.1    17    79   477   546   463   558 0.80 - Putative golgin subfamily A member 2-like protein 5  
G03801\_P             -            848 HlyD                 PF00529.15   305       2.1    6.2   9.4   1   1     0.011       4.7    5.1   9.4    65   179   365   475   342   502 0.64 - HlyD family secretion protein  
G03801\_P             -            848 IncA                 PF04156.9    191       5.3    5.3  20.8   1   1      0.37        36    2.5  20.8    76   189   356   478   323   528 0.79 - IncA protein  
G03801\_P             -            848 Laminin\_I            PF06008.9    264       5.7    4.8   7.9   1   1    0.0063       2.5    6.0   4.1    28   105   402   479   386   487 0.87 - Laminin Domain I  
G03801\_P             -            848 Laminin\_II           PF06009.7    138       1.6    7.1   5.1   1   1    0.0084       3.9    5.9   5.1    16   104   352   447   341   450 0.52 - Laminin Domain II  
G03801\_P             -            848 MAD                  PF05557.8    722     0.068    9.9   3.8   1   1   0.00049      0.12    9.1   3.8   185   312   398   516   256   545 0.60 - Mitotic checkpoint protein  
G03801\_P             -            848 MscS\_porin           PF12795.2    240       1.6    6.7  14.3   1   1     0.013       4.2    5.3  14.2    80   176   351   445   335   469 0.87 - Mechanosensitive ion channel porin domain  
G03801\_P             -            848 Myosin\_tail\_1        PF01576.14   859     0.054    9.9  21.6   1   2   2.6e-05    0.0039   13.7   8.3   156   258   346   448   340   449 0.97 - Myosin tail  
G03801\_P             -            848 Myosin\_tail\_1        PF01576.14   859     0.054    9.9  21.6   2   2     0.057       8.6    2.6   6.6   588   669   447   528   444   533 0.93 - Myosin tail  
G03801\_P             -            848 PEP-utilisers\_N      PF05524.8    123      0.27    9.7   8.8   1   3    0.0003      0.45    9.0   0.0    23    75   337   385   319   388 0.74 - PEP-utilising enzyme, N-terminal  
G03801\_P             -            848 PEP-utilisers\_N      PF05524.8    123      0.27    9.7   8.8   2   3     0.014        22    3.6   0.4    28    61   384   417   378   425 0.80 - PEP-utilising enzyme, N-terminal  
G03801\_P             -            848 PEP-utilisers\_N      PF05524.8    123      0.27    9.7   8.8   3   3      0.14   2.1e+02    0.4   2.6    24    57   423   455   414   468 0.62 - PEP-utilising enzyme, N-terminal  
G03801\_P             -            848 Reo\_sigmaC           PF04582.7    326     3e-05   22.0   4.7   1   2       4.8     1e+03   -2.7   0.0   281   308   200   227   188   234 0.86 - Reovirus sigma C capsid protein  
G03801\_P             -            848 Reo\_sigmaC           PF04582.7    326     3e-05   22.0   4.7   2   2   1.4e-07     3e-05   22.0   4.7    38   134   345   441   337   455 0.84 - Reovirus sigma C capsid protein  
G03801\_P             -            848 Spc7                 PF08317.6    326     0.006   13.9   7.6   1   1   6.3e-05     0.011   13.0   7.6   165   277   355   465   341   515 0.86 - Spc7 kinetochore protein  
G03801\_P             -            848 Syntaxin-6\_N         PF09177.6     97       3.2    6.9   7.9   1   2     0.036        24    4.1   6.2    12    69   356   421   347   451 0.62 - Syntaxin 6, N-terminal  
G03801\_P             -            848 Syntaxin-6\_N         PF09177.6     97       3.2    6.9   7.9   2   2      0.19   1.2e+02    1.8   1.7    36    62   423   449   415   501 0.74 - Syntaxin 6, N-terminal  
G03801\_P             -            848 THOC7                PF05615.8    139      0.77    8.7   7.8   1   1    0.0041       2.2    7.2   7.8    25   115   365   460   346   479 0.64 - Tho complex subunit 7  
G03801\_P             -            848 Tropomyosin          PF00261.15   237         4    5.1  18.7   1   1     0.072        17    3.0  18.5    34   137   347   450   340   509 0.67 - Tropomyosin  
G03801\_P             -            848 Tropomyosin\_1        PF12718.2    143       3.1    6.3  16.5   1   2      0.29        96    1.5   0.0    46    71   346   371   341   382 0.77 - Tropomyosin like  
G03801\_P             -            848 Tropomyosin\_1        PF12718.2    143       3.1    6.3  16.5   2   2     0.024         8    5.0  13.4     5    91   389   479   385   514 0.85 - Tropomyosin like  
G03801\_P             -            848 WEMBL                PF05701.6    522   0.00079   16.7  13.8   1   1   5.9e-06    0.0013   16.0  13.8   286   408   347   469   337   526 0.63 - Weak chloroplast movement under blue light
